# Knowledge interface co-design of a diabetes and metabolic syndrome initiative with and for Aboriginal people living on Ngarrindjeri country

**DOI:** 10.1016/j.puhip.2024.100496

**Published:** 2024-04-16

**Authors:** D. Cameron, A. Wilson, A.E. Mendham, S. Wingard, R. Kropinyeri, T. Scriven, C. Kerrigan, B. Spaeth, S. Stranks, B. Kaambwa, S. Ullah, P. Worley, C. Ryder

**Affiliations:** a Moorundi Aboriginal Community Controlled Health Service, Murray Bridge, Australia; b College of Medicine and Public Health, Flinders University, Adelaide, Australia; c Flinders Health and Medical Research Institute, Flinders University, Adelaide, Australia; d Riverland Academy of Clinical Excellence (RACE), Riverland Mallee Coorong Local Health Network, South Australia Health, Australia; e Coorong Medical Centre, Meningie, Australia; f Southern Adelaide Diabetes and Endocrine Services, South Australia Health, Adelaide, Australia; g The George Institute for Global Health, University of New South Wales, Sydney, Australia; h School of Population Health, University of New South Wales, Sydney, Australia

**Keywords:** Type 2 diabetes, Diabetes remission, Indigenous Australians, Community program, Knowledge interface methodology, Strength-based approaches

## Abstract

**Objectives:**

This research program involves two phases to identify enablers and barriers to diabetes care for Aboriginal people on Ngarrindjeri country; and co-design a strength-based metabolic syndrome and Type 2 Diabetes (T2D) remission program with the Ngarrindjeri community.

**Study design:**

A study protocol on qualitative research.

**Methods:**

The study will recruit Aboriginal people living on Ngarrindjeri country above 18 years of age with a diagnosis of metabolic syndrome or T2D. Recruitment for phases one and two will occur through the Aboriginal Health Team at the Riverland Mallee Coorong Local Health Network. The lived experiences of T2D will be explored with 10–15 Aboriginal participants, through an Aboriginal conversational technique called ‘yarning’ (60–90 min) in phase 1. Elders and senior community representatives (n = 20–30) will participate in four co-design workshops (2–4 h) in phase 2. Qualitative data will be transcribed and thematically analysed (NVivo version 12). The analysis will focus on protective factors for the Cultural Determinants of Health. Ethics approval was obtained from Aboriginal Health Research Ethics Committee in South Australia (04-22-1009), and Flinders University Human Research Ethics Committee (5847).

**Results:**

This work will be used to pilot the co-designed diabetes remission trial. Outcomes will be published in peer-reviewed journals, presented at conferences, focusing on following best practice guidelines from the Australian Institute of Aboriginal and Torres Strait Islander Studies and National Health and Medical Research Council. Research translation will occur through digital posters, manuals, and infographics.

**Conclusions:**

The findings will be summarised to all Aboriginal organisations involved in this study, along with peak bodies, stakeholders, Aboriginal Services, and interested participants.

## 1 Introduction

Type 2 diabetes (T2D) is a prevalent health challenge that impacts the health and well-being of individuals and their communities [1]. Indeed, T2D is a complex chronic disease that incorporates physical, psychological, and socioeconomic consequences that necessitates a complex and inclusive approach to mitigate its effects. A strength-based discourse around the community allows for transformative solutions that addresses the impact of T2D [2]. Accordingly, developing an environment that values communities’ voice, experiences, knowledge, and insights, can empower individuals in their health-seeking behaviour and initiate community-led projects [3]. This collaborative approach encourages stakeholders to mobilise resources, establish support networks, promote health literacy, and advocate for policies that prioritise prevention, early intervention, and holistic care. As such, engaging in robust dialogue that is centred on strengths within the community is pivotal when addressing the multifaceted impacts of T2D.

Particularly alarming is T2D impacts in Aboriginal communities in Australia, where incidence rates are three-fold higher, leading to hospitalization rates four times greater and mortality rates five times greater, than those of non-Indigenous Australians [4]. Previous strategies to address T2D risk and incidence have focused on nutrition education or health promotion in remote communities, however, prevalence rates of T2D are not declining, suggesting approaches to T2D detection, care, and management are failing Aboriginal communities [5,6]. The Australian National Diabetes Strategy has identified the need to reduce the impact of diabetes among Aboriginal people by identifying food security, healthier choices and lifestyle 7changes needed to be encouraged and/or facilitated [7]. Diabetes Australia has recommended that strategies for T2D remission be available for all Australians and acknowledges that programs need to be tailored to meet the needs of Aboriginal and Torres Strait Islander peoples [8]. These strategies include bariatric surgery, very low-calorie diets and ketogenic eating plans [8], however, accessibility to bariatric surgery is limited and adherence to a very-low calorie diet is poor. Alternatively, a ketogenic diet restricts carbohydrate intake to ensure that fat is the principal energy source. This dietary approach bears resemblance to pre-colonisation eating patterns observed among Aboriginal and Torres Strait Islander people [9]. It is conceivable that Indigenous populations may possess inherent advantages in adopting and sustaining ketogenic diets due to their alignment with Indigenous knowledge systems. By developing a feasible approach to remission through a ketogenic paradigm, we aim to improve the discourse and community support for reducing the prevalence of metabolic syndrome and T2D in the community. We will focus on co-design approaches to integrate the strengths of Aboriginal dietary knowledge with recent Western scientific knowledge on the impact of ketogenic eating plans on T2D and metabolic syndrome remission.

### 1.1 Community Co-design for type 2 diabetes remission

The Coorong Diabetes Collaborative (CDC) project was a call directly from Ngarrindjeri Elders and leaders, for a community-designed program centring on cultural determinants of health (i.e., ownership, control, reciprocity), to produce longevity for their people, to target inequitable levels of metabolic syndrome and diabetes in their community. The CDC project brings together Aboriginal and non-Indigenous researchers and clinicians across the Riverland Mallee Coorong Local Health Network (RMCLHN), Moorundi Aboriginal Community Controlled Health Service, Flinders University, and Coorong Medical Service to work with community on Country to co-design a ketogenic eating program, integrating important cultural and contextual factors as defined by the community [10]. Capacity building is an essential part of the CDC program for local Aboriginal people living on Ngarrindjeri country to implement the identification, education and monitoring approaches through upskilling and training, ensuring community ownership and control now and into the future.

As a collective, the CDC hypothesise that metabolic syndrome and diabetes remission can occur for Aboriginal people living on Ngarrindjeri country through a targeted community co-designed program. This research program involves two phases with specific aims to: (1) identify enablers and barriers to diabetes care for Aboriginal people on Ngarrindjeri country; (2) co-design a strength-based metabolic syndrome and T2D remission program with the Ngarrindjeri community. Outcomes from this work will be used to pilot the co-designed diabetes remission trial (Phase 3).

## 2 Methods and analysis

**Fig. 1:**
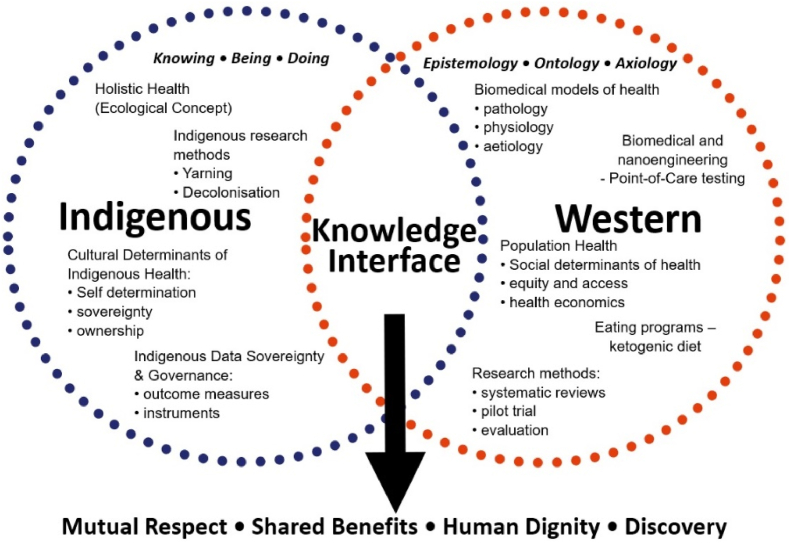
Knowledge interface between Indigenous and Western knowledges.

The project centres Indigenous knowledges through two novel methodologies (Fig. 1); 1. Knowledge interface, and 2. Strength-based approaches [2].

Knowledge interface methodology is an Indigenous methodology allowing knowledge systems, research methodologies, and methods to come together through mutual respect, shared benefits, human dignity, and discovery for new knowledge formation [2]. In this partnership, power differentials are redressed for Aboriginal and non-Aboriginal knowledges at the knowledge interface. The knowledge interface in the CDC project ensures Indigenous knowledges (knowing, being and doing) are central to research questions and the overall conceptualisation, co-design, data collection, analyses, and translation of outcomes. This will provide authentic understandings for Aboriginal communities, focusing on the cultural determinants of health. It also ensures the use of multiple approaches to investigating research questions, such as strength-based approaches and community autonomy through ownership and control.

Strength-based approaches shift deficit (negative) discourses and framing of Aboriginal health and have been used in nutrition to decolonise research and avoid perpetuating colonisation and white possessive logic [11]. Strength-based approaches are achieved by valuing and centring Aboriginal knowledges and understandings of food, nutrition, and health into research, and by recognising and actively addressing the impacts of ongoing colonisation [11]. The CDC project is grounded in strength-based approaches; evidenced through four Aboriginal CIs, strong partnerships, and support from Aboriginal CDC communities and that the CDC project has been developed from a direct request from the Ngarrindjeri community to address diabetes.

### 2.1 Project overview

**Fig. 2:**
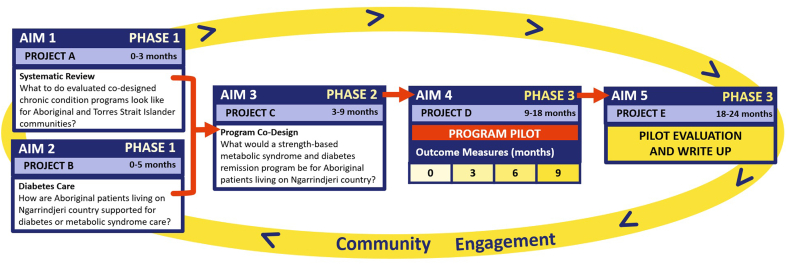
Research project overview.

The CDC project will be conducted on Ngarrindjeri country, encompassing the lower Murray, Lakes, and Coorong region of South Australia, which includes large to small rural townships (MM3-5) of the Modified Monash Model. The model measures remoteness and population size on a scale of Modified Monash (MM) category MM1 to MM7 [12]. MM1 is a major city, and MM7 is very remote. This region also aligns with the RMCLHN, in which it is estimated 700 Aboriginal people live across townships: Meningie, Murray Bridge, Tailem Bend and Raukkan (Aboriginal township), where, due to colonisation, a range of Aboriginal language groups are represented. The overall CDC study (Fig. 2) has been designed as an Aboriginal focussed community mixed-methods co-design metabolic syndrome and diabetes remission program.

### 2.2 Governance

This project draws on strong Aboriginal leadership with a core group of seven Aboriginal consumers, researchers and health professionals governing the project. This governance group meet monthly and plays a pivotal role in contributing to and approving overall study design, implementation, outcome interpretation, evaluation, translation, report writing and dissemination of the results.

### 2.3 Eligible population and recruitment

Aboriginal individuals living on Ngarrindjeri country above >18 years of age with a diagnosis of Metabolic syndrome (NCEP ATP III criteria) briefly defined by abdominal obesity, hypertension, insulin resistance, raised triglycerides, decreased HDL cholesterol), Type 2 Diabetes diagnosed by a HbA1c of ≥6.5%A1c will be eligible to participate in the study [13]. Aboriginal individuals with a diagnosis of type 1 diabetes or who are pregnant will be ineligible to participate.

Recruitment to participate in phases one and two of the study will occur through the Aboriginal Health Team at the RMCLHN. Through their work, the Aboriginal Health Team aims to empower Aboriginal people and their communities to take control of their health and make informed decisions about their well-being. They recognise the importance of cultural identity, connection to land, and community engagement in promoting positive health outcomes. Through pre-existing networks in the investigation team, potential participants will be identified and provided with an information sheet for all phases, or the study will be provided and discussed with participants. During this process potential participants will be encouraged to ask questions to clarify sections of the study. This will facilitate an informed consent process for all participants across all project phases.

### 2.4 Phase One: diabetes now

The Indigenous research method of yarning [3], a conversational technique for rich data collection, will be used to explore lived experiences of T2D or metabolic syndrome with 10–15 Aboriginal people living on Ngarrindjeri country. The yarns will be completed in an un-structured group setting to gather information through stories and lived experiences. While the yarns are relaxed and interactive, they are completed with a purpose with a defined beginning and end point [3]. Yarns will be led by two project investigators (Aboriginal researcher and non-Indigenous clinician) in group sessions on country over three locations: Meningie, Raukkan, and Murray Bridge over a period of 60–90 min. The sample size was determined through purposeful recruitment of known members in the community that meet the criteria and want to share their story.

Yarns will be recorded, transcribed and de-identified. Thematic coding of transcripts will occur through a four-part process, starting with (1) deep listening of the yarning recordings by the research coder, where important contextual points are not apparent in a transcript (i.e., use of Ngarrindjeri language, laughter) will be documented. The Aboriginal researcher present in the sessions can speak and understand Ngarrindjeri language. The participants are encouraged to express themselves in which ever language they feel most comfortable. During the transcription the Aboriginal researcher will translate back into English for thematic coding. The research coder will then (2) thematically code main themes using qualitative analysis software (NVivo version 12) with consideration of contextual points and the cultural determinants of health. During processes (1) and (2), the research coder will meet regularly meet to reflect over the developing themes (3), lastly, (4) the codes will be presented and further contextualised by the Aboriginal leadership team.

### 2.5 Phase two: diabetes the future

#### 2.5.1 Recruitment

Elders and Senior Community representatives will participate in four co-design workshops. These workshops will range from 2 to 4 h, recruitment will occur through advertisement in community-specific newsletters, flyers, and other recruitment materials (i.e., social media or email) will be used to recruit participants.

Community-led Participatory Action Research (CPAR) will be employed, where decolonising actions will occur through engaging Indigenous principles of consultation and reciprocity, and Indigenous research methods of yarning and deep listening [14]. CPAR will explore the main themes over four workshops in Table 1, with 20–30 Elders or senior community representatives of the Ngarrindjeri community. This includes Aboriginal people that are highly respected and trusted in the community with the sample size reflective of the number of Elders and senior community representatives living across these three regions. Workshops will be held on country, transcribed, and thematically analysed, focusing on protective factors for the Cultural Determinants of Health, the same coding process described in Phase One will be used. Workshops will be facilitated by community members, the investigator team, the project manager, and the leadership team.

**Table 1:** Ngarrindjeri community co-design workshops.

**WORKSHOP 1:**	Metabolic syndrome and diabetes, what are community concerns and desires for a remission program? How do we define these conditions?
**WORKSHOP 2:**	What is a ketogenic diet? What keeps people moving? What are measures of health and wellbeing?
**WORKSHOP 3:**	Critical aspects for the remission program (i.e., inclusion, focus, delivery, age) and name for the program.
**WORKSHOP 4:**	Identify implementation challenges and refine the program for Phase 3.

## 3 Ethics and dissemination

Ethics has been approved for the study through the Aboriginal Health Research Ethics Committee in South Australia (04-22-1009), and Flinders University Human Research Ethics Committee (5847), with reciprocal approval through the Riverland Mallee Coorong Local Health Network. All data will be stored on a secure, password-protected central network server at Flinders University. All paper forms will be stored in locked cupboards at Flinders University. This study will follow Flinders University's Standard Operating Procedures for data and project management. Data will be disposed of after 7 years; paper files will be securely shredded, and electronic files permanently erased. Outcomes of both phases will be published in peer-reviewed journals, presented at conferences and workshops, focusing on following best practice that aligns with the code of ethics guidelines from Australian Institute of Aboriginal and Torres Strait Islander Studies (AIATSIS) and National Health and Medical Research Council (NHMRC) national statement on ethical conduct in human research [15,16]. This process will focus on capacity building of Aboriginal researchers in the investigator team. This capacity building to foster postgraduate research opportunities tailored to recruiting prospective Aboriginal researchers, while concurrently establishing a robust platform for the development of existing Aboriginal researchers. Central to this endeavor is creating a community-engaged approach, by empowering researchers to apply their expertise directly towards improving health outcomes within Aboriginal communities. Community engagement is central to this study, for research translation through digital posters, manuals, and infographics. Infographics will be created through engagement with Ngarrindjeri artists. The research findings will be summarised to all Aboriginal organisations involved in this study, along with peak bodies, stakeholders, Aboriginal Health Services, and interested participants.

## 4 Implications

The outcomes of this study will be used to pilot the first Aboriginal co-designed metabolic syndrome and diabetes reversal program. This project differs from past programs through the use of Knowledge Interface methodology, which will bring together Indigenous Traditional knowledges and research methods from Ngarrindjeri knowledge holders (Elders, senior community representatives and leaders), Aboriginal researchers, clinicians and health professionals, with Western European Traditional knowledges and research methods from experienced clinicians, dieticians, public health researchers, point-of-care testing (POCT) and sleep experts [2]. Through mutual respect, shared benefit, human dignity and discovery, the major outcome of this project for Aboriginal people will be the co-design of the first targeted and scalable diabetes remission program for Aboriginal people on Ngarrindjeri country [10,11,17]. Research translation is a key focus for all phases of this project, allowing Aboriginal researchers, organisations, and institutions in Australia the opportunity to expand outcome use and inform co-design and diabetes research impacting on their local communities.

At the end of this project, the outcomes achieved will be.

1. A community co-design and evaluation framework that is culturally appropriate and in line with practice guidelines from Diabetes Australia in relation to T2D management for T2D remission is suitable for use by Aboriginal and Torres Strait Islander communities [18].
2. Educational materials, including digital and poster infographics for diabetes remission and the ketogenic eating plan, are suitable for Aboriginal people.
3. Empowering Aboriginal communities, through the first Aboriginal community co-designed metabolic syndrome and diabetes remission program.
4. Provide recommendations for the implementation, structure, and governance of a pilot randomized control trial.

This project will transform the treatment paradigm for Aboriginal communities for T2D and metabolic syndrome. We propose a strengths-based approach, which will flip the deficit discourse for Aboriginal people with these conditions. Examples of this deficit include the ‘non-compliance’ label too often applied by clinicians and health professionals to Aboriginal people when treatment regimens and programs are not tailored to meet their health and wellbeing requirements, as they are built from Western biomedical knowledge systems. Instead, for our project, we propose rectifying the negative impacts of previous medical-dietary advice and the sugar and flour currency of mission rations used in colonisation [11]. This project will be used to recognise the sovereignty, strength, and wisdom of Indigenous knowledges on nutrition that have existed since time immemorial, which Western European knowledges now support.

## Author contribution statement

All authors contributed to the design of the protocol. All authors contributed to writing this manuscript and reviewed the final version.

## Funding statement

Australian government's Medical Research Future Fund's (MRFF) Indigenous Health Research Fund: 2018110.

## Ethics

Ethics approval was obtained from Aboriginal Health Research Ethics Committee in South Australia (04-22-1009), and Flinders University Human Research Ethics Committee (5847).

## Declaration of competing interest

The authors declare that they have no known competing financial interests or personal relationships that could have appeared to influence the work reported in this paper.
